# Identification of sarcopenic obesity in adults undergoing orthopaedic surgery: Relationship between “a body shape index” (ABSI) and fat-free mass. A cross -sectional study

**DOI:** 10.1371/journal.pone.0269956

**Published:** 2022-06-22

**Authors:** Ana Tomažič, Boštjan Žvanut, Lilijana Vouk Grbac, Mihaela Jurdana

**Affiliations:** 1 Faculty of Health Sciences, University of Primorska, Izola, Slovenia; 2 Valdoltra Orthopaedic Hospital, Ankaran, Slovenia; Mugla Sitki Kocman Universitesi, TURKEY

## Abstract

**Background:**

Sarcopenic obesity is a condition characterised by the coexistence of low muscle mass and function (sarcopenia) and excessive fat mass (obesity). The aim of this study was to determine the prevalence of this condition in patients undergoing orthopaedic surgery by gender and type of orthopaedic surgery. In addition, this study investigated the suitability of a waist circumference-based anthropometric measure, body shape index (ABSI), for predicting sarcopenic obesity and the predictive power of ABSI for fat-free mass index (FFMI), a surrogate marker of lean body mass.

**Methods and findings:**

A cross-sectional study of overweight and obese orthopaedic patients undergoing knee or hip and spine surgery was conducted between October 2019 and March 2020 in Orthopaedic Hospital Valdoltra, Slovenia. General anthropometric parameters body mass index (BMI) and ABSI = (WC/(BMI^2/3^x height^½^) as well as body composition data (fat mass FM, fat-free mass FFM, FFMI, and the ratio FM/FFM as an index of sarcopenic obesity) were determined in 120 women (aged 66.5 ± 9.6 years) and 89 men (aged 65.5 ± 7.8 years) with overweight (25 kg/m^2^ ≤ BMI < 30 kg/m^2^) and obesity (BMI ≥ 30 kg/m^2^) by bioelectrical impedance analysis (BIA). Sarcopenic obesity phenotypes based on FM/FFM ratio > 0.80 was present in 15.3% of patients, mainly in female patients undergoing knee surgery. ABSI was significantly associated with age in all women and obese men and with waist circumference (WC) in all patients. ABSI did not correlate with BMI in women and men; however, multiple linear regression analysis showed that BMI independently predicted FFMI (R = 0.83 and 0.70, respectively, *p* < 0.001) in women and men (β-coefficients: 0.801 and 0.686, respectively) and ABSI in women only (β-coefficient: -0.104). Women with a lower ABSI had a significantly higher FFMI than the group with a higher ABSI.

**Conclusions:**

Sarcopenic obesity was most prevalent in obese women scheduled for knee surgery. In addition, ABSI, independently predicted FFMI in women and represents a significant predictor of sarcopenic obesity.

## Introduction

The increasing prevalence of obesity, in particular visceral obesity, combined with a loss of muscle mass and strength may lead to a condition called "sarcopenic obesity" [1–3]. Accurate estimation of the prevalence of sarcopenic obesity is limited due to the current lack of definitive diagnostic criteria [4, 5] and the use of different body composition assessment techniques [6, 7]. Several studies have identified sarcopenic obesity using dual-energy X-ray absorptiometry, DEXA, which is suitable for laboratory practice but requires expensive equipment which may not always be available [8]. In the clinical setting, sarcopenic obesity is defined by higher fat mass (FM) relative to fat-free mass (FFM). FM and FFM are clinically determined using bioelectrical impedance analysis (BIA), a technique available and widely implemented in clinical setting [6, 7, 9–13].

Furthermore, visceral adiposity may play an important role in the development of sarcopenic obesity, as visceral adipose tissue releases catabolic pro-inflammatory cytokines which can induce protein catabolism in skeletal muscle [8]. Furthermore, muscle unloading and physical inactivity directly promote visceral fat accumulation, leading to systemic inflammation, oxidative stress and muscle wasting [14–17].

Loss of FFM and increase in FM have a negative impact on physical abilities and contribute to morbidity and mortality in many patients [6]. Therefore, sarcopenic obesity is a condition which requires increased attention in patients undergoing orthopaedic surgery due to the accelerated loss of FFM associated with pain, mobility limitations, and obesity-related inflammation [4, 5]. In addition, the presence of sarcopenic obesity may be particularly important when surgery is indicated [18], as loss of muscle mass is associated with prolonged hospital stays, infections, non-infectious complications and disability [6, 19–21]. Some studies have also reported an association between sarcopenic obesity and therapeutic outcomes in osteoarthritis (OA) [22–25], and between sarcopenic obesity and surgical risk and recovery after joint arthroplasty [26]. It is important to note that reduced skeletal muscle mass in the older adults has been shown to have a significant impact on recovery rates and rehabilitation needs after surgery [18]. The mechanisms of sarcopenia are related to visceral obesity, and clinical studies using waist circumference (WC) as an index of abdominal fat have demonstrated an inverse relationship between abdominal fat and lean body mass [6, 17, 27]. WC is positively correlated with body mass index (BMI), a commonly used measure to assess health risks associated with obesity. However, BMI has limited ability to distinguish between muscle mass and fat accumulation and does not provide information on body shape [27].

Krakauer and colleagues [28] developed a new composite anthropometric measure, i.e. a body shape index (ABSI) based on WC and BMI. Many studies have shown that ABSI had a little correlation with height, weight or BMI, and strongly predicts morbidity, mortality [28, 29] and sarcopenic obesity [17, 27, 30–32].

Despite the negative impact of the sarcopenic obesity phenotype on the public health system and the potential consequences for orthopaedic patients, the application of practical approaches in the clinical setting represents an important step toward effective diagnosis and early prevention of potentially associated health risks. Because the latter is the ultimate goal, we hypothesise that surrogate markers may be useful for rapid screening of this condition. Therefore, the aim of this study was to investigate the prevalence of sarcopenic obesity in overweight and obese orthopaedic adult patients according to the gender and surgery type (knee, hip, or spine) as to our knowledge, little is known about its prevalence in these patients before surgery. In addition, this study aimed to evaluate the efficiency of ABSI in predicting the variability of fat-free mass index FFMI in overweight and obese orthopaedic patients. We hypothesise that ABSI is associated with sarcopenic obesity.

## Methods

### Study design and study population

In this cross-sectional study two hundred and nine overweight/obese patients (120 women; mean age 66.5 ± 9.6 years and 89 men; mean age 65.5 ± 7.8 years), Caucasian origin scheduled for total knee or hip arthroplasty or spine surgery were recruited from the Valdoltra Orthopaedic Hospital (Slovenia, EU), excluding individuals with clinically relevant comorbidities (cancer, contraindications to BIA: patients with pacemakers, implantable cardioverter defibrillators, cardiac resynchronization therapy defibrillators and active infections), patients with unstable weight within the last three months and patients with a BMI ≤ 25 kg/m^2^. Smoking was not considered an exclusion criterion. Patients were examined in the orthopaedic departments of the aforementioned institution between October 2019 and March 2020 and were subjected to anthropometric measurements and biochemical blood analysis. All procedures performed in this study were approved by the National Ethics Committee of Slovenia (Code 0120-557/2017/4) and the Ethics Committee of the Valdoltra Orthopaedic Hospital (No. 62019). All patients provided written informed consent and their rights were protected.

### Anthropometric measurements

All anthropometric measurements were taken between 7 and 9 am. The height of the subjects was measured to the nearest 0.1 cm in a standing position and without shoes, using a stadiometer (Invicta Plastic, Oadby, England). Body weight was measured (single layer of clothing, without shoes) with a precision of 0.1 kg, and the BMI (kg/m^2^) was calculated as weight (in kg) divided by the square of height (in m). WC was measured at the midpoint between the lower edge of the rib and the iliac crest using a non-stretch tape measure. All measurements were performed twice. ABSI (m^11/6^/kg^2/3^) was calculated using the following formula: WC/(BMI^2/3^x height^½^), where WC and height are expressed in metres and weight is expressed in kilograms [28].

Patients were further divided into two groups by using the median value of individual ABSI measurements as the threshold value. Subjects whose ABSI was lower than the median value were assigned to the "lower-ABSI" group, and subjects whose ABSI was higher than the median value was assigned to the “higher-ABSI” group.

### Body composition determination

In clinical setting, the ratio of FM to FFM has been introduced to identify individuals with sarcopenic obesity [6]. FM (kg) and FFM (kg) were estimated by multiple-frequency BIA using the Tanita BC 418 MA (Tanita Corporation, Arlington Heights, IL) [10]. Data were analysed using the same manufacturer’s software, which includes specific equations for different BMI ranges. BIA measurements were taken in the morning after subjects had fasted overnight. FFMI was determined as FFM (kg) divided by the square of the height in metres (m^2^). The ratio of fat mass to fat-free mass (FM/FFM) was calculated as the index of sarcopenic obesity using the following values [6]: FM/FFM ratio < 0.40 for metabolically healthy obese individuals in whom the increase in FM was minor compared to that in FFM; FM/FFM ratios between 0.40 and 0.80 for obese phenotypes in whom FM exceeded FFM, but FFM was maintained; FM/FFM ratios > 0.80 for sarcopenic obese phenotypes in whom FM was increased and FFM was reduced.

### Biochemical analysis

Serum concentrations of C-reactive protein (CRP) were determined in subgroups of women and men. Venous blood samples for biochemical analyses were collected after fasting overnight under aseptic conditions and centrifuged at 4°C in EDTA-containing plastic tubes, measured using Olympus regents and performed on an AU 680 analyser (Beckman Coulter).

### Statistical analysis

Statistical analyses were performed using IBM SPSS ver. 26. Frequencies and percentages were calculated for nominal and ordinal variables, while mean and/or median, standard deviation (SD) were calculated for numerical variables. The Kolmogorov-Smironoff and Saphiro-Wilk tests were performed to assess the normality of the distribution. This was additionally checked by assessing the histogram, skewness and kurtosis. To assess the differences in anthropometric and biochemical variables between participants with (25 kg/m^2^ ≤ BMI < 30 kg/m^2^) and (BMI ≥ 30 kg/m^2^and high/low ABSI (cut-off–median calculated separately for men and women), the independent samples t-test and non-parametric Mann-Whitney U-test were calculated. Spearman’s rank order correlation (r_s_) was calculated to assess the associations between the different variables, as the majority of variables deviated significantly from normality. Multiple linear regression was used to predict FFMI values using BMI and ABSI values. All assumptions of the multiple regression were carefully tested (e.g., multicollinearity, homoscedasticity, normal distribution of residuals, etc.). The statistical significance level was 0.05.

The a priori and post hoc statistical power calculation was performed with G *Power 3.1.9.7 [33]. The minimum required sample size (n = 31 for each gender) was calculated based on a-priory statistical power calculation for multiple linear regression (Fixed model, R^2^ deviation from zero, α = 0.05, minimal statistical power = 0.8, two predictors, assuming large effect size f^2^ = 0.35 as in comparable studies).

## Results

The anthropometric characteristics and body composition of patients are shown in Table 1. Female and male subjects were comparable in age and BMI. Body weight, WC, FFM, FFMI and ABSI were significantly higher in males, while FM/FFM ratio (ranging from 0.4 to 0.8 and > 0.8) was higher in females. CRP concentrations were not significantly different between genders.

**Table 1:** Baseline characteristics of the patients.

Parameters	Men (n = 89)	Women (n = 120)	p
Age [years]	65.5 ± 7.8	66.5 ± 9.6	NS^a^
Weight [kg]	96.2 ± 15.2	80.1 ± 12.8	< 0.001^a^
BMI [kg/m^2^]	32.4 ± 4.4	31.5 ± 4.7	NS^a^
WC [cm]	112.1 ± 12.1	102.6 ± 10.1	< 0.001^b^
FFM [kg]	63.6 ± 7.4	46.2 ± 4.9	< 0.001^a^
FFMI [kg/m^2^]	21.9 ± 2.0	19.5 ± 2.0	< 0.001^b^
FM/FFM ratio:	0.5 ± 0.1	0.7 ± 0.1	< 0.001^b^
< 0.4	20 (22.5%)	0	
[0.4–0.8]	65 (73.0%)	92 (76.7%)	
> 0.8	4 (4.5%)	28 (23.3%)	
ABSI [m^11/6^ kg^−2/3^]	0.00842 ± 0.00051	0.00818 ± 0.00044	0.001^b^
FM [kg]	32.6 ± 10.2	33.9 ± 8.7	NS^b^
CRP [mg/L]	6.1 ± 4.0	4.9 ± 1.2	NS^b^

Data are expressed means ± SD, FM/FFM ratio (< 0.4, [0.4–0.8], > 0.8) is represented as n (%), BMI—body mass index, WC—waist circumference, FFM—fat free mass, FFMI—fat–free mass index, FM/FFM ratio—ratio between fat mass and fat–free mas, ABSI—body shape index, NS- Not significant

^a^independent samples t-test

^b^Mann Whitney U test

According to the BMI standard, all patients were divided into two groups: overweight 25 kg/m^2^ ≤ BMI < 30 kg/m^2^ and obese BMI ≥ 30 kg/m^2^ to determine the prevalence of sarcopenic obesity. The anthropometric characteristics and body composition of patients according to BMI are summarised in Table 2.

**Table 2:** Anthropometric, body composition and other measured parameters of overweight and obese patients.

	BMI		
Gender	25 ≤ BMI < 30	30 ≤ BMI	p
**Men (n)**	**n = 30**	**n = 59**	
Age [years]	66.6 ± 7.3	64.9 ± 8.1	NS^a^
Body weight [kg]	81.6± 8.3	103.6 ± 12.2	< 0.001^a^
WC [cm]	101.3 ± 6.4	117.5 ± 10.5	< 0.001^b^
FFM [kg]	58.6 ± 6.3	66.2 ± 6.5	< 0.001^a^
FFMI [kg/m^2^]	20.6 ± 1.2	22.6 ± 1.9	< 0.001^b^
FM/FFM ratio	0.4 ± 0.1	0.6 ± 0.1	< 0.001^b^
< 0.4	16 (53.3%)	4 (6.8%)	
[0.4–0.8]	14 (47.7%)	51 (86.4%)	
> 0.8	0	4 (6.8%)	
ABSI [m^11/6^/kg^2/3^]	0.00843±0.00039	0.00842 ± 0.00057	NS^b^
FM [kg]	23.1± 3.2	37.5 ± 9	< 0.001^b^
CRP [mg/L]	6.0 ± 2.7	6.7 ± 4.3	NS^b^
**Women (n)**	**n = 50**	**n = 70**	
Age [years]	67.3 ± 9.7	65.9 ± 9.5	NS^b^
Body weight [kg]	70.5 ± 6.7	86.99 ± 11.7	< 0.001^a^
WC [cm]	94.8 ± 6.2	108.2 ± 8.5	< 0.001^b^
FFM [kg]	43.2 ± 3.3	48.4 ± 4.8	< 0.001^a^
FFMI [kg/m^2^]	18.1 ± 1.2	20.5 ± 1.9	< 0.001^a^
FM/FFM ratio	0.6 ± 0.1	0.8 ± 0.1	< 0.001^b^
< 0.4	0	0	
[0.4–0.8]	49 (98%)	43 (61.4%)	
> 0.8	1 (2%)	27 (36.8%)	
ABSI [m^11/6^/kg^2/3^]	0.00824±0.00046	0.00813 ± 0.00043	NS^a^
FM [kg]	27.3 ± 4.4	38.6 ± 7.9	< 0.001^a^
CRP [mg/L]	4.7 ± 0.7	5.01 ± 1.6	NS^b^

NS- Not significant, All values are mean ± SD, except distributions of FM/FFM ratio (< 0.4, [0.4–0.8], > 0.8) represented as n (%)

^a^independent samples t-test

^b^Mann Whitney U test

Both male and female obese patients showed a statistically significantly higher weight and body composition parameters (WC, FFM, FFMI, FM/FFM, FM) as compared to overweight patients. Moreover, there were no statistically significant differences in ABSI and CRP between the groups. According to the FM/FFM > 0.80, 32 of all orthopaedic patients (15.3%) had sarcopenic obesity, including 27 obese women, 4 obese men, and 1 overweight woman.

The prevalence of sarcopenic obesity varied among patients (Table 3). The highest prevalence was observed in women undergoing knee surgery, followed by spine and hip surgery. Special attention should be paid to the high prevalence of obese patients, with FM/FFM between 0.4 and 0.8 observed in all surgical groups.

**Table 3:** Prevalence of sarcopenic obesity among patients undergoing orthopaedic surgery.

Type of suregry	FM/FFM	male			female		
		n	%withinsurgery type	% within sex	n	%withinsurgery type	% within sex
Spine	< 0.4	5	22.7%	5.6%	0	0	0
	[0.4, 0.8]	16	72.7%	18.0%	19	67.9%	15.8%
	> 0.8	1	4.5%	1.1%	9	32.1%	7.5%
	**Total**	**22**	**100%**		**28**	**100%**	
Knee	< 0.4	5	15.6%	5.6%	0	0	0
	[0.4, 0.8]	25	78.1%	28.1%	33	70.2%	27.5%
	> 0.8	2	6.3%	2.2%	14	29.8%	11.7%
	**Total**	**32**	**100%**		**47**	**100%**	
Hip	< 0.4	10	28.6%	11.2%	0	0	0
	[0.4, 0.8]	24	68.6%	27.0%	40	88.9%	33.3%
	> 0.8	1	2.9%	1.1%	5	11.1%	4.2%
	**Total**	**35**	**100%**		**45**	**100%**	
**Total**		**89**		**100%**	**120**		**100.0%**

Furthermore, a Spearman rank order correlation analysis was performed to examine the relationship between anthropometric and body composition parameters in overweight and obese groups (Table 4).

**Table 4:** Relationships between anthropometric and body composition parameters according to BMI and gender.

	Weight	BMI	WC	FFM	FFMI	FM/FFM	ABSI	FM
**Men, 25 kg/m**^**2**^ **≤ BMI < 30 kg/m**^**2**^**, n = 30**								
Age	-0.147	0.028	0.186	-0.272	-0.149	0.345	0.288	0.234
Weight		0.157	0.637**	0.941**	-0.289	-0.031	0.234	0.711**
BMI			0.380*	0.124	0.699**	0.214	0.108	0.278
WC				0.530**	-0.097	0.213	0.848**	0.659**
FFM					-0.149	-0.315	0.156	0.479**
FFMI						-0.35	-0.139	-0.423*
FM/FFM							0.174	0.614**
ABSI								0.342
**Men, 30 kg/m**^**2**^ **≤ BMI, n = 59**								
Age	-0.169	0.026	0.117	-0.304*	-0.004	0.073	0.283*	-0.054
Weight		0.780**	0.767**	0.708**	0.200	0.563**	-0.170	0.859**
BMI			0.800**	0.369**	0.483**	0.627**	-0.060	0.787**
WC				0.420**	0.309*	0.572**	0.385**	0.739**
FFM					0.425**	-0.122	-0.187	0.285*
FFMI						-0.269*	0.022	-0.067
FM/FFM							0.028	0.894**
ABSI								-0.090
**Women, 25 kg/m**^**2**^ **≤ BMI < 30 kg/m**^**2**^**, n = 50**								
Age	-0.080	0.122	0.297*	-0.212	0.140	0.171	0.362**	0.013
Weight		0.491**	0.471**	0.855**	-0.257	0.535**	-0.118	0.876**
BMI			0.480**	0.301*	0.503**	0.424**	-0.070	0.551**
WC				0.1618	-0.092	0.615**	0.726**	0.622**
FFM					-0.055	0.046	-0.318*	0.519**
FFMI						-0.443**	-0.136	-0.357*
FM/FFM							0.231	0.849**
ABSI								0.058
**Women, 30 kg/m**^**2**^ **≤ BMI, n = 70**								
Age	-0.246*	-0.067	0.085	-0.395**	-0.035	0.023	0.330**	-0.168
Weight		0.735**	0.719**	0.880**	0.211	0.703**	-0.189	0.955**
BMI			0.701**	0.597**	0.683**	0.623**	-0.218	0.738**
WC				0.546**	0.252*	0.669**	0.415**	0.745**
FFM					0.340**	0.309**	-.026*	0.719**
FFMI						-0.048	-.329**	0.1124
FM/FFM							0.009	0.865**
ABSI								-0.123

All associations are presented as Spearman’s correlation coefficients (r) for men and women, splited by BMI (25 kg/m^2^ ≤ BMI<30 kg/m^2^, 30 kg/m^2^ ≤ BMI). Legend:

* p < 0.05

** p < 0.01, ABSI—body shape index, BMI—body mass index, FFMI—fat-free mass index, FM/FFM—ratio between fat mass and fat-free mass, FM- fat mass, WC—waist circumference.

In both obese groups, WC positively correlated with all measures of body composition, as well as with FM in the overweight groups, and reached a significant correlation with ABSI in all patients. In addition, a correlation between WC and FM/FFM in overweight women and FFM in overweight men was observed. In obese men and both groups of women, BMI and body composition were statistically significant for all measurements. Overall, ABSI was significantly associated with age, except in overweight men. No significant association was found between ABSI and FM/FFM ratio in all groups and ABSI and body composition parameters in both groups of men, whereas a negative correlation was found with FFM in women.

**Fig 1:**
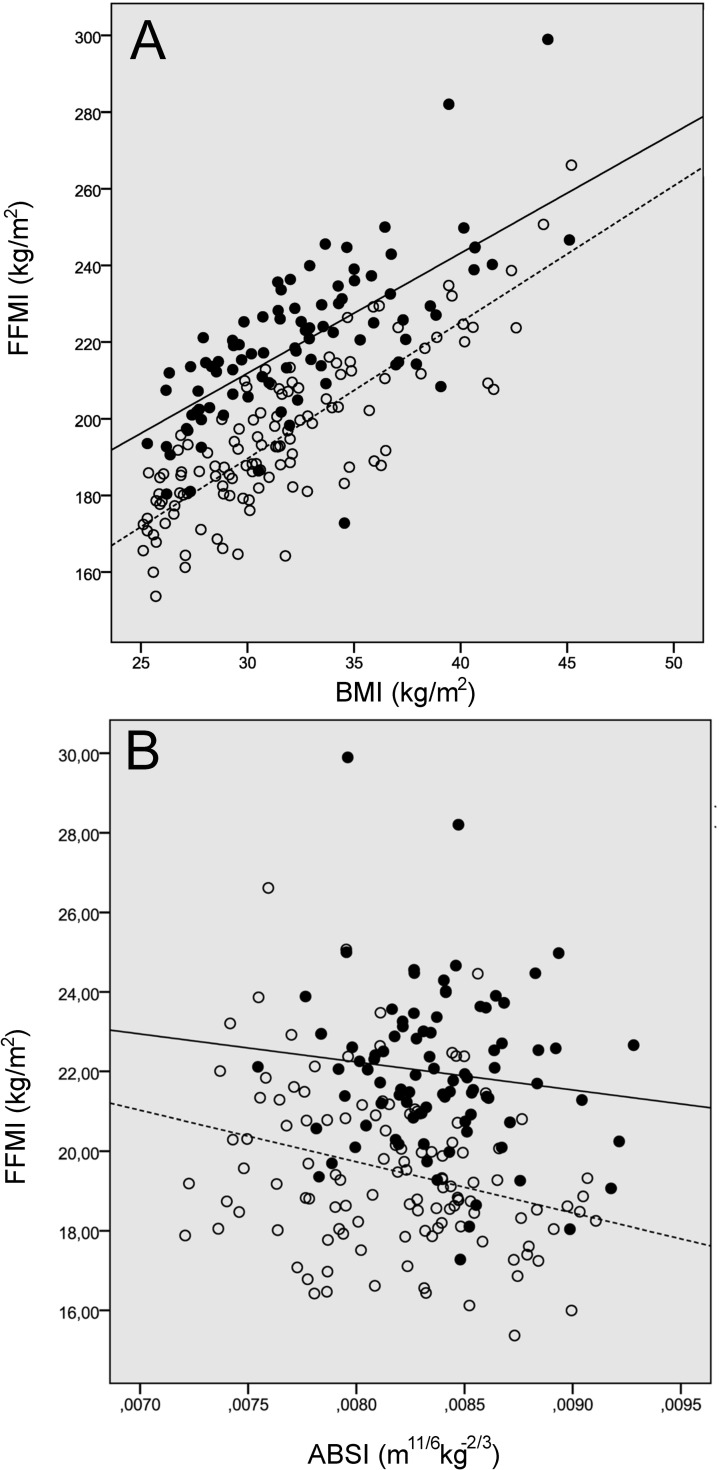
Correlations of fat-free mass index (FFMI) with body mass index (BMI) (A) and with a body shape index (ABSI) (B) in women (○) and men (●). See Table 4 for Spearman’s rank correlations and Table 5 for results of multiple regression analysis with FFMI as dependent variable and BMI and ABSI as independent variable.

Moreover, FFMI was positively associated with BMI in both sexes (Fig 1A), and negatively associated with ABSI only in obese women group (Fig 1B).

In addition, a multiple stepwise linear regression analysis was performed to identify the strongest predictors of FFMI. In both male and female patients, BMI independently predicted FFMI, whereas ABSI predicted FFMI only in the female group (Table 5).

**Table 5:** Multiple linear regression results.

Dependent variable	Women (*n* = 120)		Men (*n* = 89)	
FFMI (kg/m^2^)	*R =* 0.830		*R =* 0.703	
	*P* < 0.001		*P* < 0.001	
	1 –β = 1.000		1 –β = 0.999	
**Independent variables**	β	*P*	β	*P*
BMI (kg/m^2^)	0.801	< 0.001	0.686	< 0.001
ABSI (m^11/6^ kg^−2/3^)	-0.104	< 0.05	-0.108	NS

ABSI, a body shape index, BMI, body mass index, FFMI, fat free mass index, NS–Not Significant, 1-β—post hoc statistical power calculation.

To selectively analyse the relationship between ABSI and FFMI independent of BMI in women, women were divided into two groups using the median of each ABSI measurement as the threshold (0.00823 m^11/6^ kg^−2/3^, Table 6). Women from the “higher-ABSI” group had significantly higher mean age and WC and significantly lower BMI. In addition, FFM and FFMI were significantly lower in the “higher- ABSI” group, demonstrating the ability of ABSI to detect sarcopenia.

**Table 6:** Body parameters in female patients with lower and higher ABSI.

	ABSI women		
Variables	Lower ABSI (n = 60)	Higher ABSI (n = 60)	p
Age [years]	64.1 ± 10.2	69.0 ± 8.4	0.005^b^
Weight [kg]	82.9 ± 13.9	77.5 ± 11.2	0.046^b^
BMI [kg/m^2^]	32.5 ± 5.1	30.6 ± 4.1	0.029^a^
WC [cm]	100.3 ± 10.8	105.1 ± 8.8	0.009^a^
FFM [kg]	47.8 ± 5.2	44.7 ± 4.1	0.003^b^
FFMI [kg/m^2^]	20.0 ± 2.2	19.0 ± 1.7	0.006^a^
FM/FFM ratio	0.7 ± 0.1	0.7 ± 0.1	0.958^a^

Data are expressed as means ± SD, BMI—body mass index, WC—waist circumference, FFM—fat free mass, FFMI—fat–free mass index, FM/FFM ratio—ratio between fat mass and fat–free mas, ABSI—body shape index, Patients were stratified in two groups (“Lower-ABSI” and “Higher-ABSI”) according to the ABSI median value, (i.e., (0.00823 m^11/6^ kg^−2/3^) as threshold. Statistical analysis wasperformed using ^a^independent samples t-test; ^b^Mann Whitney U test.

## Discussion

This study investigated the prevalence of sarcopenic obesity in overweight and obese adult preoperative orthopaedic patients and evaluated the efficiency of ABSI in predicting sarcopenic obesity. The goal of ABSI is to predict disease risks that cannot be captured by BMI [34].

WC has been shown to be a valid method for assessing the risk of visceral adipose tissue accumulation [34]. We found that WC was highly and positively correlated with all body mass and composition parameters (body weight, BMI, FFM, FFMI, FM/FFM, ABSI, FM,) in the group of obese men and women (BMI ≥30 kg/m^2^). Moreover, ABSI was positively correlated with WC in all patients. Except in the overweight women group, BMI was positively correlated with body composition parameters in all patients.

The lack of correlation in the general population between ABSI, a selective marker of abdominal obesity, and BMI observed in other studies [17, 28, 29, 35] was also confirmed in the present study with no differences between genders and overweight/obese individuals.

In this study, ABSI was significantly negatively associated with FFMI in obese female patients but not in men, and consequently appears to be more relevant for predicting sarcopenic obesity in women (Fig 1). ABSI could enhance relevant information on abdominal obesity, body composition and mortality risk as previously described. A higher ABSI value indicates greater deposition of abdominal fat, which is associated with systemic inflammation and a decrease in skeletal muscle mass [28]. In our study, the association between ABSI and body composition parameters differed by gender. ABSI was negatively associated with FFM in both groups of women and with FFMI in obese women. This is likely a consequence of the different distribution of body fat between the sexes [27]. In addition, we divided the female population into two groups based on the median ABSI. The women with higher ABSI had significantly lower FFM and FFMI than the group with lower ABSI and comparable BMI. Similar results were observed in the studies by Gomez Peralta and colleagues [27] and Biolo and colleagues [17], which supports the hypothesis that abdominal fat deposition is associated with loss of skeletal muscle mass in some individuals [5, 17], leading to sarcopenic obesity.

Obesity-related sarcopenia is a progressive loss of skeletal muscle mass and function characterised by a higher FM relative to FFM [6, 27]. The results from our study confirm that ABSI is not only a marker of visceral obesity, but also an index of decreased muscle mass (i.e. sarcopenia) detected in female patients and could be useful for the initial diagnosis of sarcopenic obesity in adults’ orthopaedic patients. A positive correlation between ABSI and age is commonly reported and found in several studies [17, 22, 27, 31, 34, 36–38] and was also confirmed in all females and obese males in this study.

The direct relationship between BMI and FFMI in both sexes may reflect the anabolic efficiency of skeletal muscle loading by body weight, as isometric contraction has been shown to be an important stimulus for skeletal muscle hypertrophy [39]. The inverse relationship between abdominal fat accumulation and skeletal muscle mass loss can be explained by several mechanisms. In healthy individuals, physical inactivity is associated with increased FM, visceral fat accumulation, and decreased FFM, leading to low-grade inflammation and the development of a cluster of diseases defined by Pedersen [40] as the “diseasome of physical inactivity”.

Obesity, especially abdominal obesity, is characterised by systemic inflammation and oxidative stress, which can stimulate muscle protein breakdown and inhibit protein synthesis [15, 41, 42]. Skeletal muscle is known to be an important source of anti-inflammatory myokines, which are released during muscle contraction and play an important role in counteracting pro-inflammatory burden [43, 44]. The anti-inflammatory effect may be mediated by a reduction in visceral fat and body fat, as well as an anti-inflammatory environment through the release of myokines [45].

Furthermore, a sedentary lifestyle and/or immobility in the older adults leads to inflammation and promotes the accumulation of abdominal fat [46]. Restricting physical activity in orthopaedic patients due to pain and functional limitations or a long wait from decision to orthopaedic surgery can also lead to the same conditions. This can be a vicious cycle leading to sarcopenia and abdominal obesity.

The prevalence of sarcopenic obesity varies among different types of orthopaedic surgery and diagnostic criteria used. Previous studies examining patients undergoing orthopaedic surgery have identified more men with sarcopenic obesity [4, 47].

In our study, according to the value of FM/FFM ratio, sarcopenic obesity was most prevalent in obese women. As obesity increases, the FM/FFM ratio changes differently in the two sexes, and different mechanisms are operative in regulating muscle mass in men and women.

Suh and colleagues [24] have shown that gender-specific factors may play a role in the development of sarcopenic obesity in patients with end-stage knee OA. Indeed, high FM and low lower-extremity muscle mass were associated with the presence and severity of knee OA in women but not in men. Another study found a similar association only in women over 65 years of age [25]. As sex hormones play a central role in maintaining skeletal muscle homeostasis, the different roles of oestrogens and androgens contribute to sex differences in skeletal muscle morphology and function [48].

Our results indicate the higher frequency of sarcopenic obesity in women undergoing knee surgery. Ji and colleagues [47] reported similar findings in male orthopaedic patients; a high prevalence of sarcopenia was observed in the lower extremities. However, the obese phenotypes in this study (FM /FFM ratio between 0.4 and 0.8) occurred in all surgical groups, with a higher prevalence in knee and hip surgery in both gender, and deserve particular attention regarding the risk of developing sarcopenic obesity.

There are some limitations to our study that should be mentioned. First, the study included a relatively small number of patients and a small sample size. Further investigation involving a large number of patients is required. Second, as this study was conducted in a single orthopaedic hospital, the results could be different in other hospitals, which certainly needs to be confirmed. Third, there is a lack of additional measurements of inflammatory markers that would certainly be of interest. Finally, this study is cross-sectional. Future studies should replicate the observations and further investigate the potential use of surrogate markers for the identification of sarcopenic obesity in the clinical setting.

Based on our results we can conclude that sarcopenic obesity is more prevalent in obese women scheduled for knee surgery. In addition, ABSI, independently predicted FFMI in women and contribute to define sarcopenic obesity in females’ patients undergoing orthopaedic surgery.

## Supporting information

S1 Dataset (XLSX)

S1 File (DOCX)
