# Supplementary material for: Identification of sarcopenic obesity in adults undergoing orthopaedic surgery: Relationship between “a body shape index” (ABSI) and fat-free mass. A cross -sectional study
Source: PLoS One. 2022 Jun 22;17(6):e0269956. doi: 10.1371/journal.pone.0269956 (PMC9216617; doi:10.1371/journal.pone.0269956)
Supplement: S1 File — (DOCX) [file pone.0269956.s002.docx]

LIST OF ABBREVIATIONS

ABSI A body shape index

BIA Bioelectrical impedance analysis

BMI Body mass index

CRP C-reactive protein

DEXA Dual-energy X-ray absorptiometry

FM Fat mass

FFM Fat-free mass

FFMI Fat-free mass index

FM/FFM Ratio between fat mass and fat-free mass

OA Osteoarthritis

WC Waist circumference
